# Persistent inflammation, immunosuppression, and catabolism syndrome and sepsis in pediatric burns

**DOI:** 10.1016/j.burns.2025.107571

**Published:** 2025-06-05

**Authors:** Michael D. Santarelli, Alvin D. Jeffery, Stephanie G. Patterson, Prince J. Kannankeril, Elizabeth D. Slater, Anne L. Wagner, Ryan J. Stark

**Affiliations:** a Department of Pediatrics, Vanderbilt University Medical Center, USA; b Department of Biomedical Informatics, Vanderbilt School of Medicine, USA; c Department of Plastic Surgery, Vanderbilt University Medical Center, USA; d Department of Surgery, Vanderbilt University Medical Center, USA

**Keywords:** Persistent inflammation, immunosuppression, and catabolism syndrome, Burn-sepsis, Pediatric burn injury, Immune dysregulation, Pediatric critical care

## Abstract

**Objective:**

To determine the prevalence of persistent inflammation, immunosuppression, and catabolism syndrome (PIICS) and associated sepsis in children with burn injuries at a single, large institution over the past 25 years.

**Background:**

Despite advances in care, sepsis after burn injury continues to have a significant contribution to morbidity and mortality. This risk is compounded by an altered immune state after burn injury that can induce PIICS. While the presence of this dysregulated immunophenotype in pediatric burns is widely accepted, it remains poorly characterized.

**Methods:**

We performed a retrospective analysis of pediatric burn injuries utilizing an institutional database with de-identified patient records (1997–2023).

**Results:**

In the cohort of 287 patients, the overall prevalence of sepsis and PIICS among burn-injured children was 30 % and 15 %, respectively. The presence of inhalation injury and the total body surface area (TBSA) burned were both strongly associated with the development of sepsis and PIICS. While those with PIICS had more infections per patient compared to patients without PIICS, there was no difference in the pathogen profile between the two groups. Having PIICS itself was independently associated with the development of sepsis, and among the laboratory and clinical criteria, we found that lymphopenia had the strongest association.

**Conclusion:**

PIICS occurred in approximately 1 in 6 pediatric patients with burn injury. The development of PIICS is closely associated with the development of sepsis during hospitalization. Among the laboratory and clinical criteria, lymphopenia was most strongly associated with a septic event, suggesting that its presence should heighten concern about the development of serious infections in this vulnerable population.

## 1. Introduction

Burn injuries are an important global cause of unintentional deaths in children, with infections and sepsis reported to be a significant contributor to morbidity and mortality among burn-injured children [1–3]. Compounding this are difficulties in recognizing sepsis in this population, since the typical clinical manifestations of sepsis, such as tachycardia, tachypnea, and elevated baseline temperatures, mimic the systemic inflammatory response syndrome (SIRS) observed after burn injury [1–3]. Severe burns render patients profoundly immunosuppressed through both a compromised innate immune system via loss of the epithelial barrier, as well as a dysfunctional cellular immune response with significant leukocyte activation [1,2]. Pediatric burn-injured patients have higher infection rates than their non-burned, critically ill counterparts, with rates resembling those of other immunocompromised groups [4]. The constellation of reduced adaptive lymphocytes, chronic inflammation, and high energy needs produces an immunophenotype now recognized as persistent inflammation, immunosuppression, and catabolism syndrome (PIICS) [5,6].

While the immune dysregulation that occurs in PIICS remains poorly understood, it is likely multifactorial in nature, with several proposed mechanisms causing lymphocyte dysfunction and a hypercatabolic state. This likely begins with emergency granulopoiesis, which is commonly seen in critically ill patients in response to major injuries or systemic infection, as the body must upregulate the innate immune response [7–9]. Energy and resources are shifted towards the production of granulocytes (neutrophils, macrophages, etc.) at the expense of the production of lymphocytes [7]. With a higher number of circulating neutrophils, there is also likely an element of direct suppression of lymphocytes as CD4 + T cells have been shown to be directly suppressed in both proliferation and activity by neutrophils [9,10]. Additionally, in response to a pro-inflammatory state, production of regulatory T cells will also increase, subsequently releasing anti-inflammatory cytokines which can further suppress lymphopoiesis [9,11]. Given that the lifespan of granulocytes is relatively short, the immune system must continuously replenish the supply of these cells, devoting a lot of energy to keep up with the demands of the innate immune response. In prolonged critical illness, this process propagates a prolonged hypercatabolic state as well as ongoing suppression of lymphocytes [7,9,12, 13].

PIICS is associated with prolonged hospital stays, recurrent infections, chronic organ dysfunction, and functional decline [5,6,14]. Criteria include an intensive care unit (ICU) stay longer than 14 days, c-reactive protein (CRP) greater than 10 mg/dL, an absolute lymphocyte count (ALC) less than 800 cells/μL, and evidence of somatic protein catabolism such as weight loss greater than 10 % or albumin levels less than 3 g/dL [5,6,15]. Since the introduction of these criteria, heterogeneity regarding the definition of PIICS has developed. Several studies have suggested alterations in these criteria, including raising or lowering the cutoff values for CRP, implementing temporal associations with lab values and clinical symptoms, and the requirement of a certain number of infectious complications [5,16]. While the prevalence of PIICS is estimated to be approximately 44 % in critically ill adult populations, less is known about pediatric PIICS (pPIICS), but links between the systemic immune response and nutritional status in critically ill children have been previously described [5,17,18]. Several studies have described energy expenditure as well as the immune system’s effect on metabolic patterns in critically ill children. Specifically, stress mediators increased and nutritional markers decreased proportionally to the degree of illness severity. [17,19] Recently, we described the prevalence of pPIICS in patients who died of culture-positive sepsis in a large quaternary children’s hospital over 20 years and found that the presence of pPIICS was observed in approximately one-half of all pediatric sepsis deaths [15]. Additionally, patients with a pPIICS phenotype were more likely to have been in a surgical ICU, a finding comparable to estimates from adult populations [15,20].

Given these prior findings and the similarities between burn-injured patients and those undergoing major elective surgery, we sought to determine the overall prevalence of culture-positive sepsis and associated pPIICS in pediatric burn-injured patients within our single center over the last 25 years. Additionally, we assessed the association of certain pathogens with pPIICS as well as the individual criteria with the odds of a sepsis event to determine their overall relationships in pediatric burn injury.

## 2. Methods

### 2.1. Participants

Patients were identified within Vanderbilt University Medical Center’s (VUMC) Synthetic Derivative [21]. IRB approval was obtained from the VUMC institutional review board (#192203). Selection criteria were based on all records within the system that had an associated International Classification of Diseases (ICD) 9 or 10 code or a Current Procedural Terminology (CPT) code associated with burn injuries or care (Index e1). A manual review of individual records was performed based on the initial extraction to validate an age of 0–18 years at the time of admission. Further manual extraction from narrative data included admission and discharge dates, the burn total body surface area (TBSA) size and mechanism of the burn injury, the presence of an inhalational injury, concomitant trauma, comorbidities, and whether the hospitalization ended in mortality. Mortality was associated with the burn injury if the patient died during the initial inpatient hospitalization, however, patients who died after discharge from the primary burn-related hospitalization were not counted as a mortality. Patients admitted to the burn unit for management of desquamating skin disorders such as Stevens-Johnson Syndrome or toxic epidermal necrolysis were excluded.

### 2.2. Sepsis determination and culture data

We created custom inclusion criteria to identify patients with sepsis based on culture positivity. Patients must have had at least one of the following: a positive blood culture, a positive respiratory culture while on mechanical ventilation, or a diagnosis of sepsis within their chart (as documented in progress notes, problem lists, or ICD codes, Index e2) with a known pathogen. Wound and urine cultures were recorded but not necessarily attributed to a septic event unless indicated in the chart or were also associated with another positive culture distal to the source but overlapping in time and species. The date of positive cultures, the source of the culture data, and the type of organisms present were manually extracted and documented.

### 2.3. pPIICS determination

Patients were determined to have pPIICS if they met 4 or more of the laboratory and clinical criteria: Hospital stay of greater than 14 days, c-reactive protein (CRP) greater than 10 mg/L, absolute lymphocyte count (ALC) less than 1000 cells/μL, and evidence of somatic protein catabolism characterized by either ≥ 10 % weight loss at any point during a patient’s hospitalization based on admission weight or hypoalbuminemia defined as albumin less than 3.0 g/dL or prealbumin less than 10 g/dL, whichever was recorded. Although lymphopenia in the classic PIICS criteria is defined as ≤ 800 cells/μL, a normal lymphocyte count in children varies by age. Although lymphopenia in the classic PIICS criteria is defined as < 800 cells/μL, a normal lymphocyte count in children varies by age with the total lymphocyte count highest in the first two decades of life; decreasing between 60 % and 70 % from birth to age [14,15,20]. Given the heterogeneity of our patient’s ages and for consistency, we used the National Heart, Lung and Blood Institute’s (NHLBI) lower limit of normal in adults as our cutoff for ALC (1000 cells/μL) [22,23]. Patients with 3 of these criteria with one value missing due to the absence of data collected, were also included in the pPIICS population.

### 2.4. Statistical analysis

Percentages were used to describe categorical data with continuous data described as a median value with an interquartile range (IQR). Nonparametric categorical data were compared with chi-square or Fischer’s exact tests for univariable assessments. Nonparametric continuous data were compared with a Mann-Whitney U-test. Separate multivariable logistic regression analyses were used to test the association of age, gender, TBSA size, inhalational injury, and burn mechanism with both the development of sepsis and pPIICS. Both univariate and multivariable logistic regression analyses tested the association of hospital length of stay, lymphopenia, CRP, and somatic protein catabolism with sepsis. To analyze lab value trends over time, centered, third-order (cubic), nonlinear regression lines with 95 % likelihood CI of respective median lab values were performed for ALC, CRP, and albumin, and curves were compared using the extra-sum-of-squares F test. All data presented and analyzed adhered to the STROBE guidelines [24]. Data analysis was performed on Python 3.12 (Python Software Foundation, Fredericksburg, VA) and GraphPad Prism 10 (GraphPad Software Inc., La Jolla, CA). Results with a p-value < 0.05 were considered significant.

## 3. Results

A total of 2353 patients were identified across all age groups using the initial search criteria. Of these, a total of 287 pediatric patients (age less than or equal to 18 years old) were identified who met inclusion criteria (Figure e1). Within this cohort 89 patients (30 %) had a diagnosis of sepsis during their initial hospitalization after burn injury and 44 met the criteria for pPIICS (15 %, Table 1). Differences associated with age, race, and ethnicity were not statistically significant between groups with sepsis or pPIICS and those without. Although there was no significant difference between those with sepsis or pPIICS concerning gender, the sepsis group had 61 % males and 39 % females, similar to the male-predominant national gender distribution for burn injuries [25]. There was no significant difference in the overall prevalence of sepsis in patients seen before the chronological midpoint (2010) and those seen after (p = 0.19).

### 3.1. Sources, pathogen types, and timing of sepsis events

**Fig. 1.**
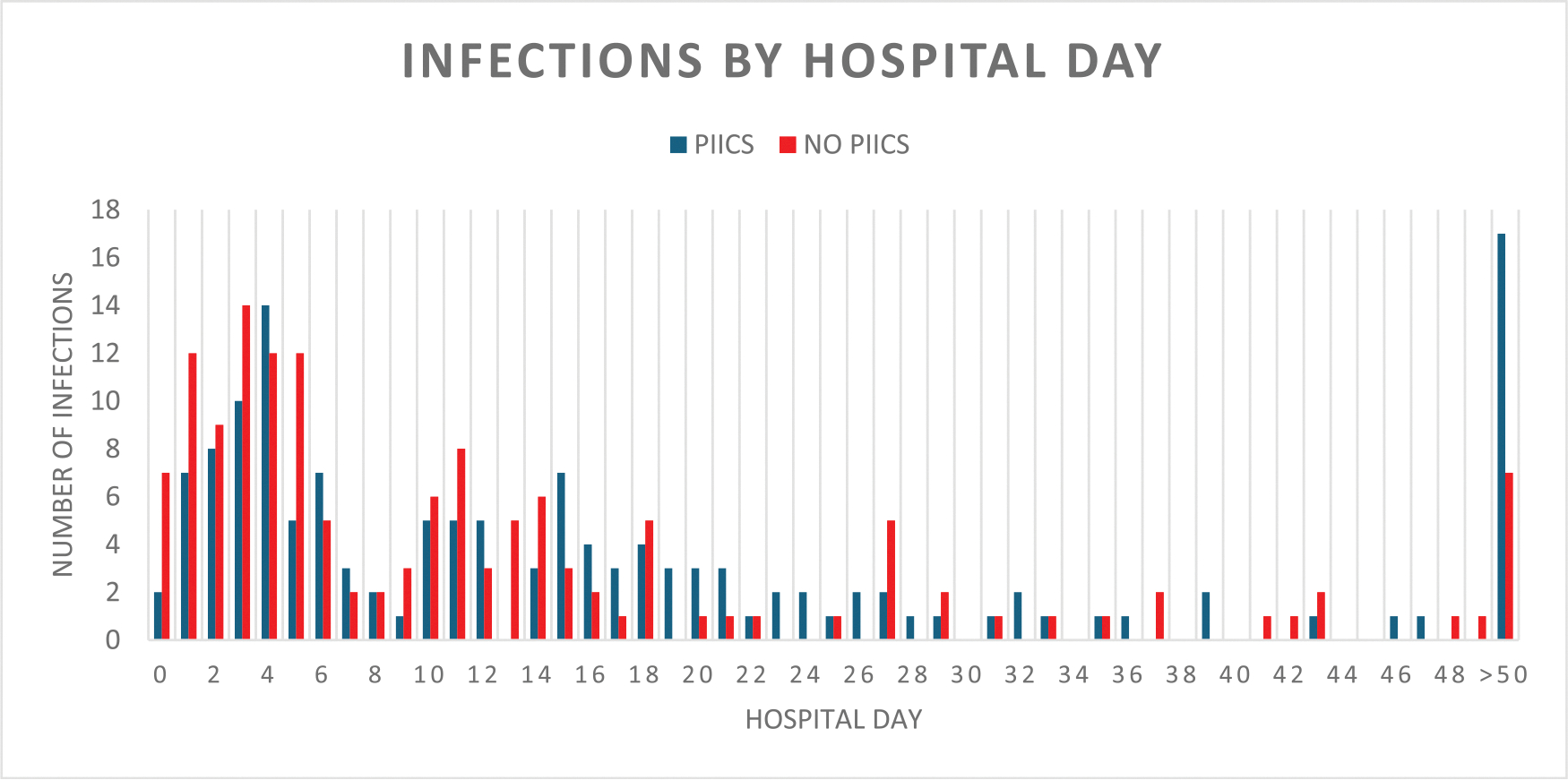
Total infections by hospital day PIICS vs no PIICS total number of infections between patients with and without piics by hospital day. abbreviations: piics-persistent inflammation, immunosuppression catabolism syndrome.

In total, 126 patients had documented culture-positive infections, 89 of which had a diagnosis of sepsis. Fifty of these patients (40 %) had positive cultures in the first 3 days of their admission and 86 (68 %) had positive cultures in the first 7 days (Fig. 1). Complete pathogen data, stratified by species and source, can be seen in Table e1. The median time to infection was the same for those with and without pPIICS at 5 days (IQR 3–10 and 2–10, respectively). Forty of the 44 patients (91 %) with pPIICS had a septic event during their hospital admission compared to 86 of the 243 patients (35 %) without pPIICS (p < 0.001; OR 18.26; 95 % CI 6.32–52.76). Patients with pPIICS incurred more infections (3.5 infections per patient in the pPIICS group vs 0.9 per patient in the non-PIICS group), but globally, there was no significant difference in the pathogen profile between patients with pPIICS and those without (Table 2).

### 3.2. pPIICS laboratory trends over time

**Fig. 2.**
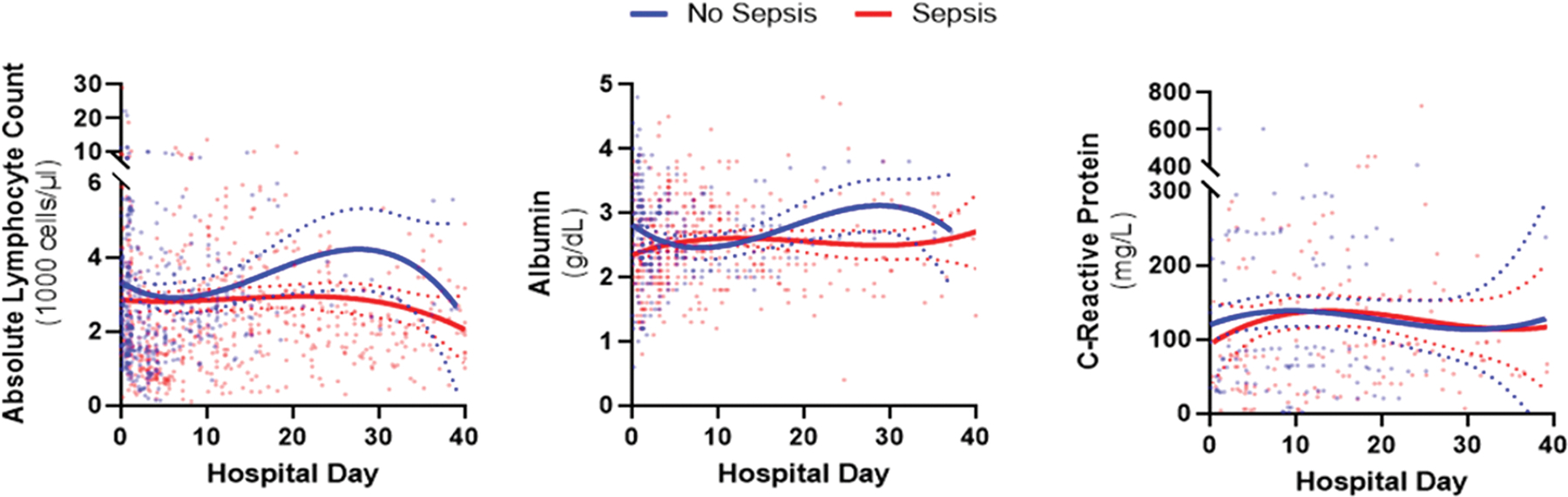
Daily lab trends of patients with sepsis vs patients without sepsis nonlinear, third-order regression lines of respective median lab value (with 95 % ci likelihood) for alc (p = 0.027; f(dfn, dfd) = 2.76 (4, 1020)), crp (p = 0.24; f(dfn, dfd) = 1.38 (4, 258)), and albumin (p = 0.006; f(dfn, dfd) = 3.64 (4, 592)) for patients post-burn with sepsis (red) and patients post-burn without sepsis (blue).

Laboratory trends of PIICS criteria over time were compared between patients with and without sepsis regarding individually obtained results for ALC, albumin, and CRP (Fig. 2). Patients with sepsis were noted to have a lower initial ALC (p = 0.027) on presentation and continued to have a flat or downward trendline for the remainder of their hospitalization. Alternatively, patients who did not develop a septic event also displayed a decrease in their initial ALC after their burn injury, but then had an increase in their ALC between days 12 and 30 of their hospitalization. Similarly, patients with sepsis had a lower initial albumin with persistent hypoalbuminemia, while patients without sepsis had an initial decrease in their albumin that also improved between days 12 and 30. There was no difference between patients with and without sepsis concerning the trend of their CRP. Additionally, using linear regression models we found there was a direct linear relationship between TBSA and albumin levels (R^2^=0.16, p < 0.01 on admission; R^2^=0.15, p < 0.01 at 24–48 h) but no linear relationship between TBSA and ALC (R^2^=0.005, p = 0.32 on admission; R^2^=0.005, p = 0.47 at 24–48 h) either on admission or after 24–48 h of care (Figure e2).

### 3.3. Analysis of burn-injury or pPIICS criteria with sepsis

Utilizing multivariable logistic regression analysis, within pediatric burn-injured patients, the presence of an inhalational injury and TBSA size had the strongest associations with a septic event (Table 3). Other characteristics, such as age, gender, and mechanism of injury, were not associated with sepsis. Additionally, those who met more pPIICS criteria were more likely to have a septic event (p < 0.001, OR 11.84; 95 % CI 5.10–27.50). The individual pPIICS criteria were also analyzed using multivariable regression analysis to determine independent associations with a septic event (Table 3). While hospital length of stay and evidence of somatic protein catabolism were both statistically significant, lymphopenia had the strongest association with the development of sepsis (OR 5.70; 95 % CI 2.27–13.01). CRP, on the other hand, had no association with the development of sepsis. In examining patients with only complete data, excluding CRP (n = 115 patients), only lymphopenia (OR 6.55; 95 % CI 2.34–20.90) and a length of stay greater than 14 days (OR 10.63; 95 % CI 2.94–50.46) were associated with sepsis development (Tables e2 and e3).

## 4. Discussion

Advancements in critical care medicine and burn-related surgical expertise have improved the survival of burn-injured patients [4–6]. The increased survivorship of the initial injury has created an emerging population of chronically critically ill patients exhibiting a paradigm of ongoing inflammation and immunosuppression after surviving a major life-threatening event. This syndrome is known as PIICS and has elements of both ongoing pro- and anti-inflammatory responses, ultimately creating an immunosuppressed state with ongoing somatic catabolism [5,6,15]. Few PIICS studies have focused on burn-injured patients or the pediatric population. In this single-center retrospective study of 25 years’ worth of data collection, we identified 287 pediatric burn-injured patients. 31 % of burn-injured children at our center had a septic event, and 15 % met the criteria for pPIICS. We further found that the presence of inhalational injury and TBSA size had the strongest association with both sepsis and the development of pPIICS. Amongst all the pPIICS criteria, lymphopenia had the strongest association with sepsis. Taken in summation, these data would suggest that increased severity of burn injury, as assessed by either inhalational injury, TBSA, or both, drives significant depression in the adaptive immune system, setting up an increased risk in the development of pPIICS and ultimately, sepsis.

Severe burn injury is associated with a catabolic state with alterations in adaptive immunity, both critical criteria of the PIICS phenotype [26]. Despite this, there have been no specific characterizations of the prevalence or risk factors associated with PIICS in pediatric burn injury. Our results showed that the presence of inhalational injury, followed by TBSA size, had the strongest association with pPIICS. This is consistent with what others have identified as the main risk factors for sepsis and mortality in pediatric burn patients, but their association with PIICS has not yet been documented [1,27–29]. Likewise, PIICS has been associated with increased nosocomial infections, but little data exists regarding which type of pathogens these patients become infected with. Studies regarding the etiology of infections in immunosuppressed patients have suggested that most are secondary to gram-negative bacteria or fungal infections [30]. Our prior analysis of patients who died of pediatric sepsis found no difference in gram-positive or negative organisms but did find an increased mortality rate in fungal-related sepsis [15]. Given their baseline immunosuppressed state, burn patients are already at risk for infections with fungal species and multidrug-resistant organisms [1–3]. Our findings suggest the immunosuppression associated with PIICS renders these patients more susceptible to infections of any etiology. Indeed, despite similarities in culture profiles, we found that lymphopenia was the most important driver of predicting a sepsis event, irrespective of the other PIICS criteria. There have been long-standing links established between lymphopenia and poor outcomes [9]. Specifically, lymphopenia has been associated with multi-organ dysfunction syndrome (MODS), immune paralysis, increased risk of secondary infections, as well as death [9, 31–33]. While here we identified lymphopenia’s importance and association with negative outcomes in pediatric burn injury, this only furthers the growing body of literature detailing the importance of recognizing lymphopenia in critical illness.

Despite the growing body of literature surrounding PIICS, the primary challenge remains in what to do once the phenotype is recognized, given its insidious onset [5]. A single therapeutic agent is unlikely to provide an adequate remedy, and instead, multimodal therapies may be required to address both the dysregulated immune system of these individuals and the ongoing somatic catabolism [5,34]. As mentioned previously, a hypermetabolic state with secondary profound protein catabolism is a common finding among burn patients with ≥ 30 % TBSA [5,35,36]. Several anabolic supplements have been studied in this population, including human growth hormone, the anabolic steroid oxandrolone, and propranolol [5,37–40]. Oxandrolone was once a common therapy, however, it was withdrawn from the market by the Food and Drug Administration in late 2023 [38,39,41]. As a potential alternative, human growth hormone has been found to decrease muscle catabolism and osteopenia in pediatric burn patients [5,37]. In addition, propranolol has also been found to decrease burn-related proteolysis as well as increase muscle anabolism [5,40]. To mitigate the immunosuppression, granulocyte-macrophage colony-stimulating factor (GM-CSF) may be one potential therapy in the reversal of immune dysregulation as data from adult and pediatric studies have found that GM-CSF may reverse immunoparalysis and immunosuppression associated with sepsis [30,42–44]. Studies regarding the use of interleukin 7 (IL-7) have also demonstrated that supplementation may be able to reverse sepsis-associated lymphopenia [45]. In addition, interferon-gamma (IFN-γ) has been tried in limited situations to improve immune dysfunction [46].

Other strategies that may be useful in the prevention of PIICS are similar to some that are employed in the prevention and treatment of the hypermetabolic state associated with burn injury. These include early treatment of the underlying etiology, limiting secondary insults, ensuring adequate nutrition, and early mobility [47,48]. Early excisional treatment and wound closure have been shown to decrease hospital length of stay as well as infectious complications. Additionally, time to primary surgical excision and grafting has been shown to be an important predictor of the hypermetabolic response [35]. While aggressive treatment of the primary insult is paramount, limiting secondary insults is arguably equally important. Complications such as additional operations or hospital-acquired infections can further potentiate the exaggerated inflammatory response, with further suppression of lymphocytes. Additionally, early enteral nutrition has long been viewed as beneficial in critically ill patients, but alone, this is unlikely to prevent or support the hypercatabolic state of PIICS [47,49]. Several nutritional strategies have been proposed, including a high protein diet, arginine and leucine supplementation, the addition of antioxidants, and tight glycemic control [47,48]. Although a recent randomized controlled trial showed that supplementation of oral glutamine did not improve survival after severe burn injury, this has not been explicitly studied in PIICS [50]. Given our continued limited mechanistic understanding of why PIICS develops, therapies and preventative strategies to effectively combat it remain elusive.

There are several limitations to our study given its design. First, the identification of patients depended on the assignment of accurate billing codes, which can vary based on providers [51]. Second, given that several patients were missing data related to our PIICS criteria, including documentation of weights and laboratory data, we may have underestimated the prevalence of PIICS in this population. We used strict parameters for our inclusion into the PIICS cohort as patients needed to have all four criteria met or meet three criteria with one criterion not documented, further increasing the likelihood of underestimating the true prevalence of PIICS. Third, we also possibly underestimated the prevalence of sepsis given the way information is processed for this database, where culture data had to be manually extracted from patient charts. In addition, we were unable to account for external modifiers, such as antibiotic usage, that could have altered the number of sepsis events. Fourth, we are unable to infer laboratory cut-off values for the prediction of the development of infections or pPIICS given the overall waxing and waning nature of PIICS and the amount of missing laboratory data surrounding a septic event. Fifth, since our study spanned 25 years, the long time span may have led to differences in diagnosis and treatment standards, potentially affecting which patients would be included. Sixth, given that this was a single-center study at a large quaternary care children’s hospital, there is a significant potential for bias and confounding factors that we could not control. Lastly, the overall generalizability is limited given the retrospective nature and the fact that all of this data came from a single study.

## 5. Conclusion

Utilizing a database of pediatric burn-injured patients admitted to a single institution over the last 25 years, we found an overall prevalence of pPIICS of approximately 1 in 6 patients. As expected, inhalation injury and TBSA burned had strong associations with the development of sepsis and pPIICS. Patients with pPIICS had more infections per patient compared to those without pPIICS, but there was no major difference in the pathogen profiles. Finally, although the PIICS criteria of lymphopenia, albumin, and length of stay were all significant in their association with the development of sepsis, lymphopenia had the strongest association. Further investigation of pPIICS, specifically into novel, prospective identifiable risk factors of its development, requires additional pursuit.

## Supplementary Material

Supplement

## Figures and Tables

**Table 1:** Characteristics of patients with or without pPIICS or sepsis.

	Total (N = 287, %)	PIICS Post Burn (N = 44,%)	No PIICS Post Burn (N = 243, %)	P value	Septic Post Burn (N = 89, %)	Non-Septic Post Burn (N = 198, %)	P value

**Gender**				0.13			0.69
Male	180 (63)	23 (50)	157 (65)		54 (61)	126 (64)	
Female	107 (37)	21 (50)	86 (35)		35 (39)	72 (36)	
**Age**	5 (2–12)	6 (2–12)	5 (2–12)	0.44	5 (2–13)	5 (2–12)	0.76
**Hospital Length of Stay**	14 (6–33)	44 (25–77)	12 (4–25)	< 0.001	39 (15–67)	11 (4–19)	< 0.001
**Race**				0.41			0.88
White	202 (71)	29 (66)	173 (71)		64 (72)	138 (70)	
Black	50 (17)	9 (20)	41 (17)		16 (18)	34 (17)	
Other	35 (12)	6 (14)	29 (12)		9 (12)	26 (13)	
**Ethnicity**				0.12			0.44
Hispanic/Latino	18 (6)	5 (11)	13 (5)		7 (8)	11 (6)	
Non-Hispanic/Latino	269 (94)	39 (89)	230 (95)		82 (92)	187 (94)	
**Presence of Inhalational Injury**	61 (21)	15 (34)	46 (19)		37 (42)	24 (12)	
**Mechanism of Burn**				0.16			0.12
Flame	178 (62)	28 (64)	150 (62)		63 (71)	115 (58)	
Scald	100 (35)	13 (30)	87 (36)		24 (27)	76 (38)	
Other^a^	9 (3)	3 (6)	6 (2)		2 (2)	7 (4)	
**TBSA Size**				0.002			< 0.001
< 20 %	64 (22)	4 (9)	60 (25)		9 (10)	55 (28)	
20 %-49 %	187 (65)	28 (64)	159 (65)		58 (65)	129 (65)	
> 50 %	36 (13)	12 (27)	24 (10)		22 (25)	14 (7)	
**Co-trauma on Admission**	20 (7)	3 (7)	17 (7)	0.97	8 (9)	12 (6)	0.38
**Comorbidities**							
Neurologic	11 (4)	1 (2)	10 (4)	0.56	4 (5)	7 (4)	0.7
Respiratory	10 (4)	0 (0)	10 (4)	0.17	2 (2)	8 (4)	0.44
Cardiovascular	5 (2)	0 (0)	5 (2)	0.35	0 (0)	5 (2)	0.13
Renal, Hepatic or Gastrointestinal	0 (0)	0 (0)	0 (0)	N/A	0 (0)	0 (0)	N/A
Hematologic or Oncologic	2 (1)	0 (0)	2 (1)	0.55	0 (0)	2 (1)	0.34
Endocrine	0 (0)	0 (0)	0 (0)	N/A	0 (0)	0 (0)	N/A
Psychiatric	15 (5)	3 (7)	12 (5)	0.61	4 (5)	11 (6)	0.71
Social	9 (3)	3 (7)	6 (3)	0.13	4 (5)	5 (3)	0.38
Tobacco Use	6 (2)	2 (5)	4 (2)	0.72	3 (3)	3 (2)	0.31
Drug or Alcohol Use	6 (2)	3 (7)	3 (1)	0.02	3 (3)	3 (2)	0.31

Data presented as number (%) or median (interquartile range)

Abbreviations: PIICS-Persistent Inflammation, Immunosuppression Catabolism Syndrome, TBSA-Total Body Surface Area

a -“Other” burn mechanisms included friction, chemical and high voltage electrical burns

**Table 2:** Number of infections in patients with pPIICS vs patients without pPIICS.

Organism Isolated	PIICS Isolates (N = 152)	No PIICS Isolates (N = 220)	Total Isolates (N = 372)	OR	95 % CI	P value

**Gram Positive Organisms**						
Staphylococcus aureus	33	52	85	0.896	0.55–1.47	0.71
Streptococcus pneumoniae	13	10	23	1.96	0.84–4.60	0.13
Enterococcus faecalis	7	8	15	1.28	0.45–3.61	0.79
Streptococcus pyogenes	2	3	5	0.96	0.16–5.84	1.00
Enterococcus NOS	1	2	3	0.72	0.06–8.04	1.00
Staphylococcus coagulase negative	7	7	14	1.47	0.50–4.28	0.58
Enterococcus faecium	1	1	2	1.45	0.09–23.4	1.00
Streptococcus agalactiae	3	1	4	4.41	0.45–42.8	0.31
Gram Positive species NOS	0	2	2	0.29	0.01–6.02	0.52
Total Gram positive Isolates	67	89	156	0.753	0.49–1.17	0.22
**Gram Negative Organisms**						
Pseudomonas aeruginosa	17	22	39	1.13	0.58–2.21	0.73
Enterobacter cloacae	9	12	21	1.09	0.45–2.66	1.00
Escherichia coli	7	11	18	0.92	0.35–2.42	1.00
Haemophilus influenzae	7	14	21	0.71	0.28–1.80	0.50
Acinetobacter baumannii	2	7	9	0.41	0.08–1.98	0.32
Acinetobacter NOS	3	2	5	0.20	0.02–1.66	0.15
Pseudomonas Species NOS	3	6	9	0.72	0.18–2.92	0.74
Stenotrophomonas maltophilia	3	4	7	1.09	0.24–4.93	1.00
Klebsiella pnuemoniae	2	3	5	0.96	0.16–5.84	1.00
Pseudomonas putida	2	2	4	1.45	0.20–10.4	1.00
Enterobacter Species NOS	1	7	8	0.20	0.02–1.66	0.15
Klebsiella oxytoca	1	1	2	1.45	0.09–23.4	1.00
Moraxella catarrhalis	0	6	6	0.11	0.001–1.9	0.09
Serratia marcescens	0	3	3	0.21	0.01–4.03	0.28
Serratia Species NOS	0	2	2	0.29	0.01–6.02	0.52
Gram Negative Species NOS	3	6	9	0.72	0.18–2.95	0.74
Total Gram Negative Isolates	66	110	176	0.77	0.51–1.16	0.25
**Fungal Species**						
Candida albicans	11	11	22	1.47	0.62–3.45	0.38
Candida parapsilosis	3	2	5	2.20	0.36–13.3	0.40
Candida tropicalis	1	0	1	4.37	0.18–108	0.41
Candida Species NOS	3	7	10	0.61	0.16–2.41	0.54
Mucor Species NOS	1	1	2	1.45	0.1–23.4	1.00
Total Fungal Isolates	19	21	40	1.35	0.70–2.62	0.40

Abbreviations: PIICS-Persistent Inflammation, Immunosuppression Catabolism Syndrome, NOS- Not otherwise specified, OR- Odds Ratio, CI- Confidence Interval Isolates with only one positive result in either category and not included in the table above were as follows: Bacillus Species NOS, Staphylococcus lugdunenesis, Abiotrophia defective, Acinetobacter calsoceticus, Acinetobacter haemolyticus, Acinetobacter lwoffi, Aeromonas hydrophila, Enterobacter aerogenes, Morganella morganii, Citrobacter koseri, Providencia stuartii,

An additional five isolates of Sars-Cov-2 and one isolate of parapertussis from nasopharyngeal swabs all from the “No pPIICS” group not listed above

**Table 3:** Association of patient characteristics or PIICS criteria with the development of sepsis.

Variable	Odds Ratio	95 % Confidence Interval	P Value

Gender	1.04	0.56–1.94	0.9
Age	0.99	0.93–1.05	0.69
TBSA Size	3.02	1.31–6.97	0.01
Inhalational Injury	5.31	2.48–11.38	< 0.001
Burn Mechanism (Flame as Reference)			
Scald	0.93	0.40–2.16	0.60
Other	0.33	0.04–2.47	0.30
PIICS Criteria			
LOS> 14 days	5.0	2.29–10.91	< 0.001
Lymphopenia	5.70	2.27–13.01	< 0.001
Evidence of Somatic Catabolism^a^	3.38	1.58–7.20	0.002
CRP	0.00	0.00 to Infinite	N/A

Separate multivariable logistic regression analysis of patient characteristics and PIICS criteria with the development of sepsis.

Evidence of somatic protein catabolism defined as presence of either Albumin ≤ 3.0 g/dL, OR prealbumin ≤ 10 g/dL OR Weight loss > 10 % from admission weight at any point during admission

Abbreviations: PIICS-Persistent Inflammation, Immunosuppression Catabolism Syndrome, TBSA- Total Body Surface Area, LOS- Length of Stay

## Data Availability

Original data elements are available from the authors upon request.

## Appendix A. Supporting information

Supplementary data associated with this article can be found in the online version at doi:10.1016/j.burns.2025.107571.

## Abbreviation:

ALC: absolute lymphocyte count
Alb: albumin
CRP: C-reactive protein
ICU: intensive care unit
LOS: length of stay
PIICS: persistent inflammation, immunosuppression, and catabolism syndrome in adults
pPIICS: pediatric persistent inflammation, immunosuppression, and catabolism syndrome
SIRS: systemic inflammatory response syndrome
SJS: Stevens-Johnson syndrome
TBSA: total body surface area
TEN: toxic epidermal necrolysis
